# Past and future of a century old *Citrus tristeza virus* collection: a California citrus germplasm tale

**DOI:** 10.3389/fmicb.2013.00366

**Published:** 2013-12-10

**Authors:** Jinbo Wang, Orhan Bozan, Sun-Jung Kwon, Tyler Dang, Tavia Rucker, Raymond K. Yokomi, Richard F. Lee, Svetlana Y. Folimonova, Robert R. Krueger, John Bash, Greg Greer, James Diaz, Ramon Serna, Georgios Vidalakis

**Affiliations:** ^1^Department of Plant Pathology and Microbiology, University of CaliforniaRiverside, USA; ^2^Department of Plant Protection, University of ÇukurovaAdana, Turkey; ^3^United States Department of Agriculture-Agricultural Research Service, San Joaquin Valley Agricultural Sciences CenterParlier, CA, USA; ^4^United States Department of Agriculture-Agricultural Research Service, National Clonal Germplasm Repository for Citrus and DatesRiverside, CA, USA; ^5^Department of Plant Pathology, University of FloridaGainesville, Florida, USA

**Keywords:** bioindexing, diversity, stem pitting, seedling yellows, virus exclusion

## Abstract

*Citrus tristeza virus* (CTV) isolates collected from citrus germplasm, dooryard and field trees in California from 1914 have been maintained *in planta* under quarantine in the Citrus Clonal Protection Program (CCPP), Riverside, California. This collection, therefore, represents populations of CTV isolates obtained over time and space in California. To determine CTV genetic diversity in this context, genotypes of CTV isolates from the CCPP collection were characterized using multiple molecular markers (MMM). Genotypes T30, VT, and T36 were found at high frequencies with T30 and T30+VT genotypes being the most abundant. The MMM analysis did not identify T3 and B165/T68 genotypes; however, biological and phylogenetic analysis suggested some relationships of CCPP CTV isolates with these two genotypes. Phylogenetic analysis of the CTV coat protein (CP) gene sequences classified the tested isolates into seven distinct clades. Five clades were in association with the standard CTV genotypes T30, T36, T3, VT, and B165/T68. The remaining two identified clades were not related to any standard CTV genotypes. Spatiotemporal analysis indicated a trend of reduced genotype and phylogenetic diversity as well as virulence from southern California (SC) at early (1907–1957) in comparison to that of central California (CC) isolates collected from later (1957–2009) time periods. CTV biological characterization also indicated a reduced number and less virulent stem pitting (SP) CTV isolates compared to seedling yellows isolates introduced to California. This data provides a historical insight of the introduction, movement, and genetic diversity of CTV in California and provides genetic and biological information useful for CTV quarantine, eradication, and disease management strategies such as CTV-SP cross protection.

## Introduction

*Citrus tristeza virus* (CTV) isolates from citrus materials introduced in California between 1914 and 2009 have been maintained *in planta* under quarantine in the Citrus Clonal Protection Program (CCPP) at the University of California, Riverside (UCR). Therefore, this unique assemblage of CTV isolates has preserved the genetic profile of CTV collected over time and space in California and provides unique spatiotemporal materials to examine CTV genetic and biological attributes that are relevant for current management and detection strategies.

The CTV isolates in the CCPP collection represent two distinct CTV periods in California. The first CTV period can be defined from the late 1800's, when citrus was first introduced in the state, until 1957, when the Citrus Variety Improvement Program (CVIP), the precursor of the CCPP, was established. For example, in the 1870s, the Parent Washington navel and other citrus varieties were introduced from Brazil and Far East, respectively, for commercial use. In 1907, the Citrus Experiment Station (CES, UCR's precursor) was established and citrus materials began to be introduced systematically to California for experimental use. At that time the viral nature of tristeza (quick decline) disease was unknown and the graft-transmissible nature of citrus diseases was not discovered until much later (i.e., 1933, citrus psorosis). As a result, these early commercial and CES citrus introductions were performed without any specific disease screening (Hiltabrand, 1959; Wallace and Drake, 1959; Hodgson, 1967; Soost et al., 1977; Calavan et al., 1978; Wallace, 1978; Roistacher et al., 1981; Lawton and Weathers, 1989; Kahn et al., 2001). Scientific developments at the CES and Brazil between 1946 and 1951 (Meneghini, 1946; Wallace and Drake, 1951) suggested a viral etiology for tristeza (quick decline) disease, a hypothesis later supported by observation of virus-like particles associated with diseased plants (Kitajima et al., 1964). Indexing on Mexican lime (*Citrus aurantifolia* Christm. Swing.) showed a CTV association with the tristeza quick decline (QD) epidemics which decimated citrus on sour orange rootstock in southern California (SC) at that time (Fawcett and Wallace, 1946; Bar-Joseph et al., 1981; Roistacher, 1995; Agranovsky, 1996; Karasev, 2000; Lee and Bar-Joseph, 2000; Gottwald et al., 2002; Garnsey et al., 2005). Based on the knowledge obtained with tristeza (quick decline), as well as parallel discoveries on bioindexing of citrus virus and virus-like pathogens, the CVIP was inaugurated at UCR in 1957. From that point on, systematic indexing of citrus introductions to California led to the discovery of various CTV isolates and the establishment the collection used in this study (Weathers and Calavan, 1959; Calavan et al., 1978).

The second CTV California period represented in the CCPP collection is from 1957–2009. During this period, citrus plantings in central California (CC) were developed using QD-tolerant trifoliate (*Poncirus trifoliata* L. Raf.) and trifoliate-hybrid rootstocks. Since southern vs. CC citrus-growing regions are separated by the Tehachapi Mountains, which range from 1.5–2.1 km in elevation and stretches for a distance of 60–80 km, this separation isolated a revitalized citrus industry from the QD-affected areas of SC (Calavan et al., 1978; Barnier et al., 2010). Furthermore, in 1963, a CTV eradication program, managed by the CC Tristeza Eradication Agency (CCTEA), was established in CC where ~200,000 acres or 76% of the California citrus industry is located today (Gottwald et al., 2002; Usda-Nass, 2012). In 2009 and after localized high CTV incidence in some survey areas, the Citrus Pest Detection Program (CPDP) of the CCTEA adopted a CTV suppression program focused on a selective removal of CTV positive trees from CC based on serological and genotypic criteria (Permar et al., 1990; Yokomi and Deborde, 2005; Barnier et al., 2010; Yokomi et al., 2011a,b).

The CTV isolates from the two spatiotemporal periods described above are represented in the CCPP collection as derived from: (i) CES and CCPP introductions from exotic sources, (ii) citrus trees of various ages that became naturally infected with strains of CTV by vectors from urban landscapes and commercial citrus groves primarily in SC; (iii) interceptions by the CPDP of citrus illegally propagated or topworked using CTV infected budwood as well as isolates being spread by aphid vectors in CC.

In this study, genotypes of 48 CTV isolates, representing approximately 90% of the CCPP CTV collection, were characterized using multiple molecular markers (MMM) assays (Hilf et al., 2005; Yokomi and Deborde, 2005; Moreno et al., 2008; Folimonova et al., 2010; Roy et al., 2010; Roy and Brlansky, 2010). The MMM genotype characterization was complimented by CTV phylogenetic analysis of the major coat protein (CP) gene. Finally, the genetic data of specific CTV isolates were correlated with spatiotemporal information and biological activity on indicator plants (Garnsey et al., 1987, 1991; Polek et al., 2005). These findings serve as a reference for CTV genetic profiles collected over the past century in California and provide a valuable database for CTV management strategies such as CTV strain differentiation and mild strain cross-protection as well as regulatory actions.

## Materials and methods

### CTV sources

CTV isolates were obtained from the CCPP *in planta* Citrus Pathogen Collection at Riverside, CA. Source plants were graft-inoculated Madam Vinous sweet orange (*C. sinensis* L.) and represent isolates from imported citrus propagations from abroad as well as field sources collected in California over the past century. The CCPP CTV were separated in categories according to the following; location: SC; CC; excluded from California (XC) i.e., intercepted in California and eliminated so there was no field spread; CTV period: 1907–1957 (1), 1957–2009 (2); type of original source: A = CES and CCPP introductions, B = infected trees from urban landscapes or commercial groves; and C = interceptions by the CPDP (Table 1).

**Table 1:** ***Citrus tristeza virus*(CTV) isolates in the Citrus Clonal Protection Program (CCPP) collection, University of California, Riverside**.

**Name**	**Isolation**	**Origin**	**Location**	**Period**	**Type**	**Genotype**	**CP Gene**	**CP Gene**	**Biological**
	**year**						**GenBank**	**phylogenetic**	**characterization**
								**clade**	**score sum**
SY550	1963	P. R. China	SC	2	A	VT	^##^798	7	18
SY551	1967/1917^a^	Riverside, CA	SC	1	A	T30+T36	^##^822	3	15
SY553	1960/1917	Riverside, CA	SC	1	A	VT+T36	^##^823	3	10.7
SY554	1963	Riverside, CA	SC	2	A	VT	^##^799	5	14
SY555	1971	Riverside, CA	SC	2	A	VT	^##^800	7	10
SY556	1972	Waiakea, Hawaii	XC	2	A	VT	^##^801	5	18.9
SY557	1971	Waiakea, Hawaii	XC	2	A	VT	^##^802	5	21.8
SY558	1968/1914	Honolulu, Hawaii	SC	1	A	T30+VT+T36	^##^826	7	22
SY560	1978/1914	Riverside, CA	SC	1	A	T30+VT	^##^813	5	16.7
SY561	1978/1918	Riverside, CA	SC	1	A	T30	^##^793	1	14
SY563	1976/1914	Brazil: Bahia	SC	1	A	T30	^##^794	1	17.6
SY565	1978/1914	Australia	SC	1	A	VT	^##^803	4	15.5
SY566	1978/1914	Honolulu, Hawaii	SC	1	A	VT	^##^804	5	23.1
SY568	1978/1961	Riverside, CA	SC	2	A	T30+VT	^##^814	6	28.3
SY575	1980	San Bernardino, CA	SC	2	B	T30+VT	^##^815	5	16
SY576	1982	San Bernardino, CA	SC	2	B	T30+VT	^##^816	5	15
SY577	1979/1914	Miami, Florida	SC	1	A	T30+VT	^##^817	5	23.3
SY578	1979/1948	Riverside, CA	SC	1	A	T30	^##^795	1	14
SY579	1979	Orange, CA	SC	2	B	T30	^##^796	1	13.5
SY580	1980/1963	Riverside, CA	SC	2	A	T30+VT	^##^818	7	7
SY581	1983	Riverside, CA	SC	2	A	T30+VT	^##^819	1	nt
SY583	1979/1914	Florida	SC	1	A	VT+T36	^##^824	7	25
SY584	2009	Argentina: Tucuman	XC	2	A	T30+VT	^##^820	7	nt
T19	1975	Riverside, CA	SC	2	A	T30+VT	^##^805	1	nt
T500	1968	Riverside, CA	SC	2	A	T30	^##^779	1	4
T505	1971	Central CA	CC	2	C	T30	^##^780	1	7
T506	1971	Ventura, CA	SC	2	B	T30	^##^781	1	2.5
T508	1971	Ventura, CA	SC	2	B	T30	^##^782	1	6
T509	1972	Orange, CA	SC	2	B	T30+VT	^##^806	1	nt
T510	1972	San Bernardino, CA	SC	2	B	T30+VT	^##^807	1	7
T511	1972	Ventura, CA	SC	2	B	T30	^##^783	1	4
T514	1974	Tulare, CA	CC	2	C	T30	^##^784	1	6
T515	1977	Calaveras, CA	CC	2	C	T30+VT	^##^808	1	8.5
T517	1979	Orange, CA	SC	2	B	T30	^##^785	1	nt
T518	1979	Orange, CA	SC	2	B	T30+VT	^##^809	1	nt
T519	1978	Riverside, CA	SC	2	A	T30+VT	^##^810	6	nt
T520	1978	Tulare, CA	CC	2	C	T30	^##^786	1	nt
T521	1980	Orange, CA	SC	2	B	T30+VT	^##^811	1	nt
T522	1981	Ventura, CA	SC	2	B	T30	^##^787	1	nt
T524	1981	Tulare, CA	CC	2	C	VT	^##^797	7	nt
T525	1975	Orange, CA	SC	2	B	T30+T36	^##^821	2	3.5
T528	1990	Tulare, CA	CC	2	C	T30	^##^788	1	nt
T529	1990	Tulare, CA	CC	2	C	T30	^##^789	1	nt
T530	1990	Tulare, CA	CC	2	C	T30	^##^790	1	nt
T531	1992	Florida	XC	2	A	T30	^##^791	1	nt
T532	1996	Australia: Victoria	XC	2	A	T30+VT	^##^812	5	nt
T534	2008	San Diego, CA	SC	2	C	T30	^##^792	1	nt
T535	2000	Japan	XC	2	C	T30+VT+T36	^##^825	3	25

a *The year of the original CTV record is reported if it is different from the isolation year; SC, CC, and XC: South, Central, and Excluded, California, respectively; 1, first and 2, second CTV California periods; A, Citrus Experiment Station/CCPP introductions; B, urban/commercial areas infected trees; and C, Citrus Pest Detection Program interceptions; CP, coat protein*,

## KC841-GenBank contain additional notes on original host and history, nt: not tested.

### Multiple molecular markers (MMM) analysis

The MMM genotype analysis of CTV was performed using reverse transcription polymerase chain reaction (RT-PCR) with specific primers for the genotypes T30, VT, T36, T3, and B165/T68, as previously described (Hilf et al., 2005; Roy and Brlansky, 2010; Roy et al., 2010) (Table 2). Total RNA from ~0.2 g of bark tissue was extracted using the Spectrum Plant Total RNA kit (Sigma, Saint Louis, Missouri, USA) according to the manufacturer's instructions and eluted in 30 μl of RNase-free water. RT-PCR was performed using AMV Reverse Transcriptase Kit and GoTaq Hot Start Green Master Mix Kit (Promega, Madison, WI, USA) or Qiagen One-Step RT-PCR Kit (Qiagen, Germantown, MD, USA) using proper positive and negative controls as previously described (Hilf et al., 2005; Sharma et al., 2011). The RT-PCR products were analyzed using electrophoresis on 1% agarose gel and visualized over a UV transilluminator after ethidium bromide staining. All MMM reactions were repeated twice and at least two RT-PCR amplicons, per CTV isolate, were sequenced in order to verify homology with the corresponding CTV genome regions. Sequence analysis was performed with ClustalX (1.81), BioEdit (7.0.5.3), and GeneDoc (2.7.000) software (Nicholas and Nicolas, 1997; Thompson et al., 1997; Hall, 1999). The frequencies of the CTV genotypes were calculated as the sum of genotype counts in single, double, and triple mixtures.

**Table 2:** **Multiple molecular markers (MMM) and primers**.

**MMM^a^**	**Reference sequences**	**Primer names**	**Primer sequences**
CP-U	AF260651	CP-U-F-16054-16075	CWTGAGCRCTGCTTTAAGGGTC
		CP-U-R-16836-16814	GATGAAACTCCACCATCCCGATA
T30-5'-H	AF260651	T30-5'-F-6-26	CGATTCAAATTCACCCGTATC
		T30-5'-R-600-580	TAGTTTCGCAACACGCCTGCG
T30K17-H	AF260651	T30K17-F-4848-4870	GTTGTCGCGCCTAAAGTTCGGCA
		T30K17-R-5256-5235	TATGACATCAAAAATAGCTGAA
T30POL-H	AF260651	T30POL-F-10772-10791	GATGCTAGCGATGGTCAAAT
		T30POL-R-11467-11448	CTCAGCTCGCTTTCTCACAT
T30-R	AF260651	T30-F-588-613	TGTTGCGAAACTAGTTGACCCTACTG
		T30-R-793-769	TAGTGGGCAGAGTGCCAAAAGAGAT
VT-5'-H	U56902	VT-5'-F-1-22	AATTTCTCAAATTCACCCGTAC
		VT-5'-R-492-472	CTTCGCCTTGGCAATGGACTT
VTK17-H	U56902	VTK17-F-4824-4846	GTTGTCGCGCTTTAAGTTCGGTA
		VTK17-R-5232-5211	TACGACGTTAAAAATGGCTGAA
VTPOL-H	U56902	VTPOL-F-10745-10764	GACGCTAGCGATGGTCAAGC
		VTPOL-R-11440-11421	CTCGGCTCGCTTTCTTACGT
VT-R	U56902	VT-F-1945-1972	TTTGAAAATGGTGATGATTTCGCCGTCA
		VT-R-2246-2222	GACACCGGAACTGCYTGAACAGAAT
T36-5'-H	U16304	T36-5'-F-1-20	AATTTCACAAATTCAACCTG
		T36-5'-R-500-481	CTTTGCCTGACGGAGGGACC
T36K17-H	U16304	T36K17-F-4871-4892	GTTTTCTCGTTTGAAGCGGAAA
		T36K17-R-5279-5258	CAACACATCAAAAATAGCTAGT
T36POL-H	U16304	T36POL-F-10797-10816	TGACGCTAACGACGATAACG
		T36POL-R-11511-11490	ACCCTCGGCTTGTTTTCTTATG
T36-R	U16304	T36-F-1775-1799	TTCCCTAGGTCGGATCCCGAGTATA
		T36-R-2610-2585	CAAACCGGGAAGTGACACACTTGTTA
T3K17-H	EU857538	T3K17-F-4846-4867	GTTATCACGCCTAAAGTTTGGT
		T3K17-R-5254-5233	CATGACATCGAAGATAGCCGAA
T3-R	EU857538	T3-F-4846-4873	GTTATCACGCCTAAAGTTTGGTACCACT^c^
		T3-R-5254-5231	CATGACATCGAAGATAGCCGAAGC^c^
B165/T68-R^b^	EU076703	B165/T68-F-1885-1912	GTCAAGATTTTGATGATTTGTGCCACTC
		B165/T68-R-2633-2607	AAAATGCACTGTAACAAGACCCGACTC

a Two MMM methodologies were developed independently by Hilf et al. (2005)(-H) and Roy and Brlansky (2010)(-R) and Roy et al. (2010). CP-U: Coat protein universal primer was developed in this study and was used as positive internal control for CTV detection.

b Genotypes B165 (EU076703) and T68 (JQ965169) represent the same genotype (Folimonova et al., 2010; Roy and Brlansky, 2010).

c Extra nucleotide sequences of T3-R compared to the T3K17-H sequences are bold and underlined.

### Coat protein gene phylogenetic analysis

A set of primers for universal CTV detection was designed from the genomic region of the CP gene (CP-Universal, CP-U) using the Primer 3 software (Rozen and Skaletsky, 2000) (Table 2). The RT-PCR products were purified using Zymo Research DNA Clean and Concentrator Kit (Zymo Research, Irvine, CA, USA), and then sequenced directly using the CP-U forward and reverse primers as previously described (Hajeri et al., 2011). For each of the 48 CTV isolates, the CP-U RT-PCR amplified products were sequenced in order to obtain the complete sequence of CTV CP gene (Table 2). Consensus sequences were assembled using DNA Dragon software (http://www.dna-dragon.com) with 2–3 × coverage per strand yielding a sequence of 672 nt in length corresponding to the CP gene. The CTV CP gene sequences acquired were deposited in GenBank (GenBank accession numbers KC841779-KC841826).

Thirty-four full-length CTV genome and CP gene sequences produced at previous studies available in the GenBank were analyzed phylogenetically (data not shown). Subsequently, eleven GenBank CTV sequences representatives of the identified phylogenetic clades, namely, T30 (AF260651), T36 (U16304), NUagA (AB046398), NZ-M16 (EU857538), VT (U56902), B165/T68 (EU076703), NZRB-M12 (FJ525431), A18 (JQ798289), SY568 (AF001623), HA16-5 (GQ454870), and T3 (KC525952) were selected for analysis with the CTV isolates in this study.

All topologies were reconstructed with neighbor-joining, maximum parsimony and maximum likelihood methods using MEGA5.1 software. The confidence level in tree topology was examined using bootstrap with 10,000 replicates (Tamura et al., 2011). The topologies from the three phylogenetic methods were similar so only the neighbor-joining phylogenic tree is presented.

### Biological characterization

CTV biological characterization was performed between 1970 and 2012 by graft-inoculation of Mexican lime [*C. aurantifolia* (Christm.) Swing.], Duncan grapefruit (*C. paradisi* Macf.), Eureka or Lisbon lemon (*C. limon*L. Burm.f.), sour orange (*C. aurantium* L.), and Madam Vinous or Pineapple sweet orange seedlings as previously described (Roistacher, 1991; Garnsey et al., 2005; Polek et al., 2005). CTV isolates were inoculated using at minimum six seedlings of each plant indicator, including negative and positive controls. Indicator plants were maintained under standard greenhouse conditions (24–28°C day and 17–21°C night temperatures), infection was confirmed by enzyme-linked immunosorbent assay (ELISA) and symptoms were rated 6–9 months post-inoculation. Symptoms were evaluated on a 0–5 scale (0 = negative; 1 = very mild; 2 = mild; 3 = moderate; 4 = severe; 5 = very severe) over several replicated experiments and the average score for each indicator among different experiments was used (Table 4). Finally, the sum score of the seedling yellows (SY) (0–15) and SP (0–10) indicators as well as the total score for all indicators (0–30) for each isolate were calculated and used for statistical analysis.

The biological characterization data were tested for normality using the Kolmogorov–Smirnov test. Normally distributed data were analyzed by One Way ANOVA, otherwise, the non-parametric Kruskal–Wallis test on ranks (ANOVA on Ranks) was conducted. Statistically significant differences among means were identified by the Holm-Sidak method. Normality test, ANOVA, and tests of significance were performed at *p* < 0.05 using the Sigma Plot 11.00 software (Copyright© 2008 Systat Software, Inc.; San Jose, CA, USA).

## Results

### CTV genotypes determination

The MMM analysis is presented in Table 3. The identification of the T30 and VT genotypes in single infections were in general agreement (25 out of 26) between MMM-H and MMM-R (one isolate reacted differently). In contrast, seven CTV isolates reacted differently for MMM-H and MMM-R in the mixtures of T30 and VT genotypes (Table 3). The T36 genotype was identified only in mixtures with T30 and VT genotypes (Table 3).

**Table 3:**
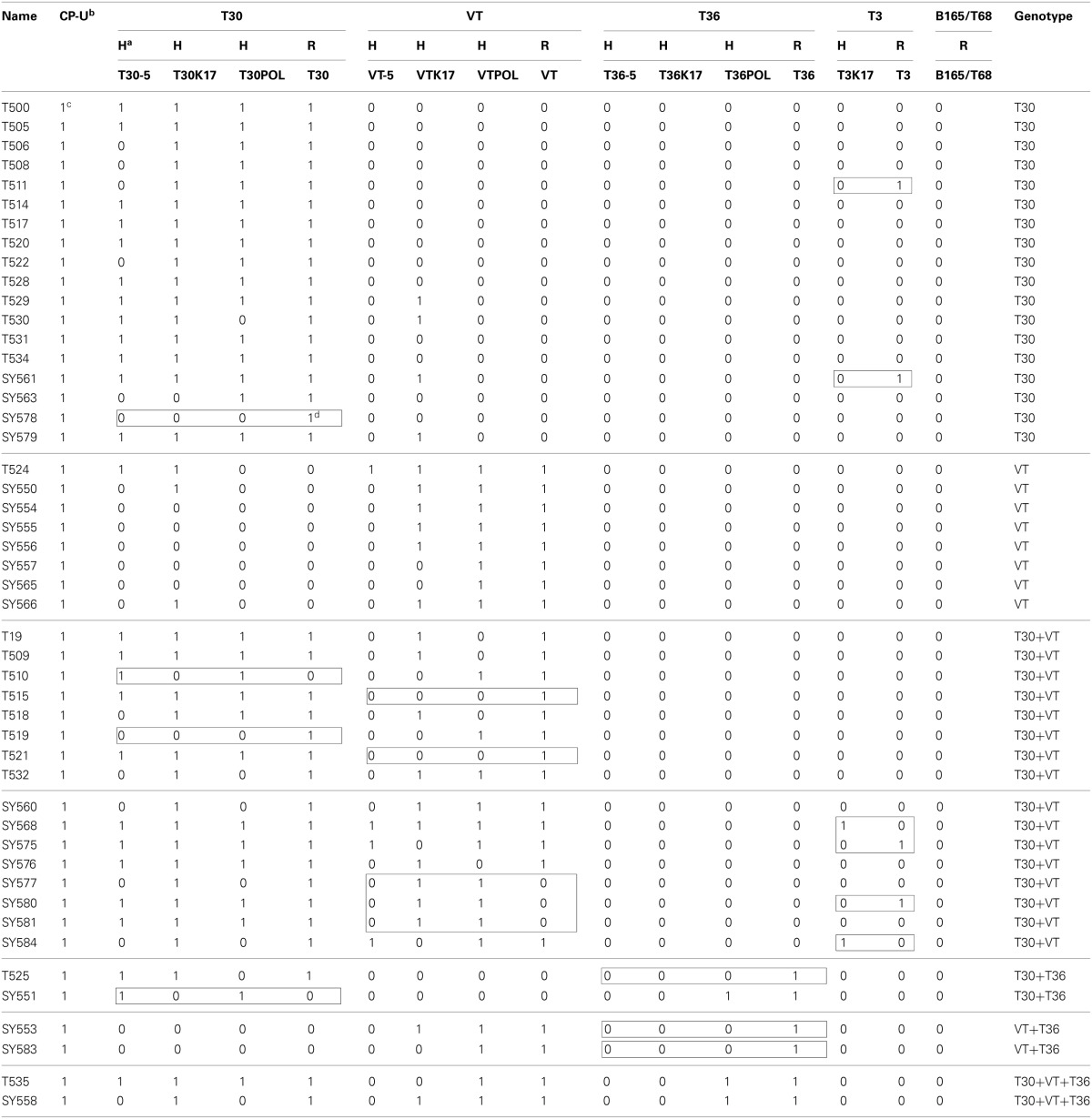
**Multiple molecular markers (MMM) analysis of the *Citrus tristeza virus*(CTV) isolates in the Citrus Clonal Protection Program collection**.

^a^Two MMM methodologies were developed independently by Hilf et al. (2005) (H) and Roy and Brlansky (2010) (R) and Roy et al. (2010). ^b^CP-U: Coat protein universal primer was used as positive internal control for CTV detection. c0 and1: negative and positive reaction, respectively. dBoxed areas indicate result differences between MMM-H and MMM-R.

Genotype T3 markers developed by Roy et al. (2010) are located in the exact same CTV genome area and contain the identical sequence of that of Hilf et al. (2005) except having a few extra nucleotides at the 3' end of primers (Table 2). Interestingly enough, the T3 markers reacted differently for the CTV isolates tested by MMM-H versus MMM-R. There was a general agreement (42 out of 48) on isolates identified as non-T3 but the two MMM systems identified six different isolates as T3 genotype (Table 3). Sequence analysis of the T3 MMM-H and MMM-R PCR products showed 85–89% similarity with the k17 region of ORF 1a of the newly characterized T3 representative (Harper, 2013). The highest sequence similarity (95–97%) was observed with the k17 region of the Indian isolates BAN-1 (AY285670), BAN-2 (AY285668), and B226 (AY285669) which have been reported as various mixtures of T30, VT, T36, and T3 genotypes (Roy and Brlansky, 2004). Hence, the determination of T3 genotypes in the CCPP CTV collection was inconclusive and the T3 genotype was excluded from further analysis.

**Figure 1:**
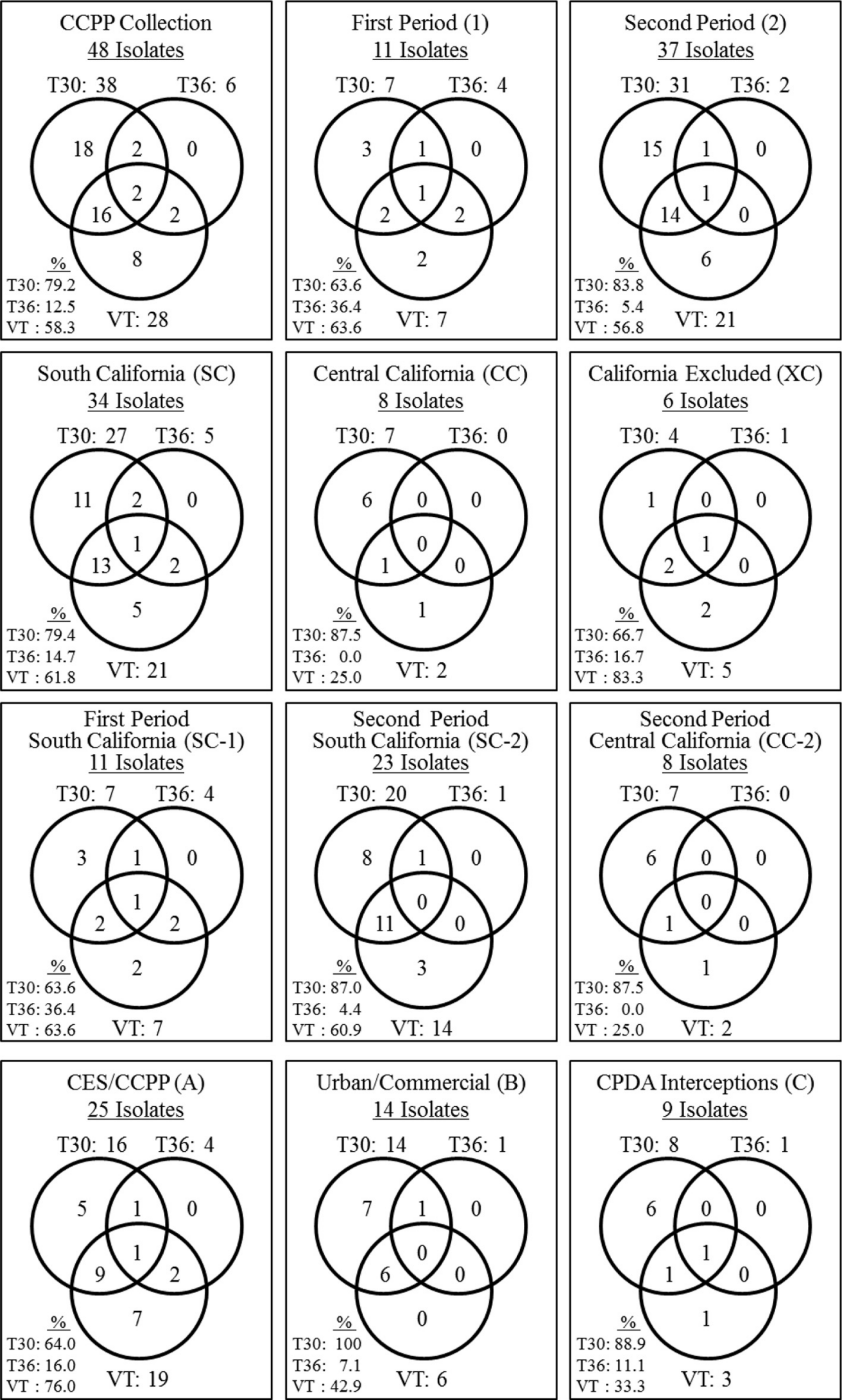
**Summary of the genotype frequencies of the *Citrus tristeza virus* (CTV) isolates in the Citrus Clonal Protection Program (CCPP) collection and specific CTV genotype frequencies for different isolation periods (first and second), locations (South, Central, and Excluded, California), and types of CTV sources (Citrus Experiment Station-CES/CCPP introductions, urban/commercial areas, and Citrus Pest Detection Program (CPDP) interceptions)**. The second period Excluded California genotype frequencies were identical to the Excluded California frequencies thus, is not presented separately. The CTV genotypes frequencies were calculated as the sum of genotype counts in single, double, and triple mixtures.

Eighteen CTV isolates contained the single genotype T30 (37.5%) and eight isolates contained the VT (16.7%) genotype. No CTV isolate was found solely with the T36 genotype (Figure 1). The remaining 22 CTV isolates contained mixtures of two or three of T30, T36, and VT genotypes (45.8%). The most common genotype mixture was T30+VT identified in 16 isolates (33.3%). The genotype mixtures of T30+T36, VT+T36, and T30+VT+T36 represented 4.2% of the CTV isolates (Figure 1).

### CTV genotypes spatiotemporal analysis

The CTV genotype frequencies and spatiotemporal distribution is presented in Figure 1. T30 and VT were identified at the same frequency for period 1. In period 2, the T30 genotype frequency increased while VT and T36 frequencies decreased (Figure 1). The T30 genotype frequency was similar for SC and CC (i.e., 79.4% and 87.5%, respectively). On the contrary, the VT genotype frequency was reduced to less than half in CC in comparison to SC while the T36 genotype was not detected in the CC isolates. All three genotypes were identified amongst the six California excluded (XC) isolates intercepted in the state but eliminated before field spread (Figure 1).

The diversity of the SC-2 CTV genotypes was reduced in comparison to the SC-1. Two genotype mixtures identified in SC-1 (VT+T36 and T30+VT+T36) were not identified in SC-2. In addition, the genotype frequency for T30 increased and T36 decreased in SC-2 in comparison to SC-1 (Figure 1). T30 and VT were the only genotypes identified in CC-2. The CC-2 genotypic diversity was not as big as the SC-1. For example, three genotype mixtures identified in SC-1 (T30+T36, VT+T36 and T30+VT+T36) were not identified in CC-2 while VT and T36 frequencies were reduced (Figure 1). Finally, the T30 genotype frequency was stable (~87%) for SC-2 and CC-2, however, VT and T36 frequencies were reduced in CC-2. All three genotypes were identified in CTV isolates originated from CES/CCPP introductions, urban/commercial groves, and CPDP interceptions (Figure 1).

### CTV CP gene phylogenetic and spatiotemporal analysis

**Figure 2:**
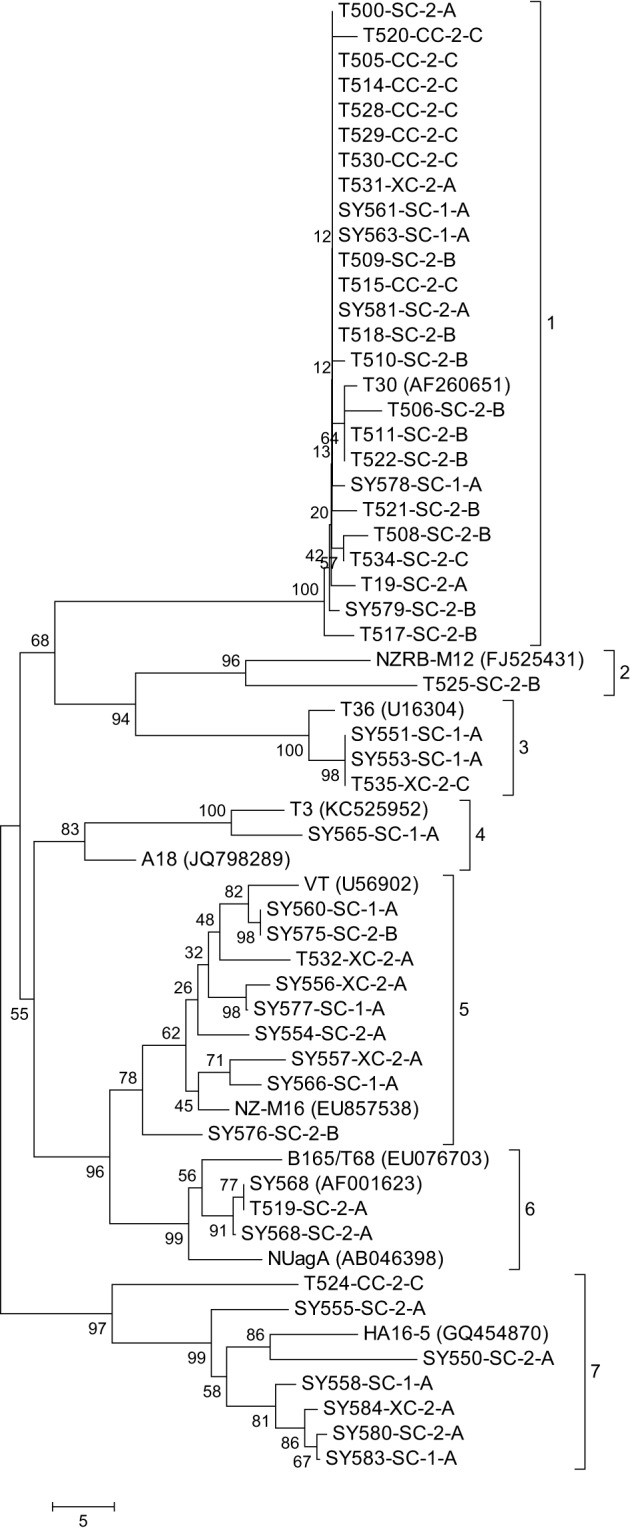
**Neighbor joining tree of the complete sequence of the major coat protein gene of the *Citrus tristeza virus* (CTV) isolates in the Citrus Clonal Protection Program (CCPP) collection including 11 representative CTV isolates previously deposited in GenBank (accession numbers in parenthesis)**. Bootstrap values (10,000 replicates) are shown next to the branches and identified phylogenetic clades are labeled 1–7. The name of the CTV isolate is followed by the geographic location; SC, CC, and XC: South, Central, and Excluded, California, respectively; the CTV isolation period: 1: early and 2: second; and the types of isolations: A, Citrus Experiment Station/CCPP introductions; B, urban/commercial areas infected trees; and C, Citrus Pest Detection Program (CPDP) interceptions.

GenBank contains a plethora of CTV sequences. In order to select appropriate CTV representative accessions for a meaningful analysis with the CCPP CTV isolates, GenBank CTV accessions were analyzed phylogenetically on their own. The analysis identified five distinct clades associated with the T30, T36, VT, B165/T68, and T3 CTV genotypes and three additional clades with isolates not related to any standard CTV genotype (data not shown). The CP gene sequence of 11 GenBank CTV accessions representing the eight identified phylogenetic clades was selected for analysis with the 48 CCPP CTV isolates in this study (Figure 2).

The 48 CCPP CTV isolates were clustered into seven distinct clades (Figure 2). SC and XC isolates were present in seven and four clades, respectively. In contrast, CC isolates were limited in two clades. California CTV isolates from periods 1 and 2 were well-distributed and present in five and six clades, respectively. CTV isolates from type A were present in six clades while types B and C were limited in three clades (Figure 2).

The majority of the CCPP CTV isolates (34 out of 48) clustered with representatives of the T30 and VT genotypes (clade 1 and 5). A total of six CCPP CTV isolates clustered with the representatives of T36, T3, and B165/T68 representatives (clade 3, 4, and 6). The remaining isolates clustered with the non-standard CTV genotypes NZRB-M12 (clade 2) and HA16-5 (clade 7) (Figure 2). It is worth noting here that NZRB-M12 and HA16-5 were strongly associated (bootstrap >85%) with CCPP CTV isolates of T30+T36 and VT genotype, respectively. (Vives et al., 2005; Harper et al., 2010; Melzer et al., 2010; Harper, 2013).

### CTV biological characterization, spatiotemporal and genotypic analysis

Results of biological indexing per isolate are presented in Table 4. Mexican lime reacted with all CTV isolates tested; three isolates did not induce SY; and 16 isolates did not induce SP reactions. Four CTV isolates reached the maximum SY sum score of 15 while nine additional isolates scored 12 and above. In contrast, no CTV isolate reached the maximum SP sum score of ten and only two isolates scored above seven (Table 4).

**Table 4:** **Biological characterization reactions of the *Citrus tristeza virus* (CTV) isolates in the Citrus Clonal Protection Program collection**.

**Name**	**ML**	**Seedling yellows**				**Stem pitting**		
		**SO**	**Le**	**GF**	**Sum**	**GF**	**SW**	**Sum**
SY550	5	5	3	5	13	0	0	0
SY551	2	3	3	5	11	0	2	2
SY553	1	3.2	3	3.5	9.7	0	0	0
SY554	3	2.5	5	3.5	11	0	0	0
SY555	5	0	0	0	0	5	0	5
SY556	4	1	1.8	4.4	7.2	4.1	3.6	7.7
SY557	2	5	5	4.8	14.8	0	5	5
SY558	5	4.2	4.9	3.6	12.7	0	4.3	4.3
SY560	3	4.7	4.4	4.6	13.7	0	0	0
SY561	5	0	5	4	9	0	0	0
SY563	4	4.6	4.4	4.6	13.6	0	0	0
SY565	3	4	3.7	4.8	12.5	0	0	0
SY566	4	5	5	5	15	0	4.1	4.1
SY568	5	5	5	5	15	3.3	5	8.3
SY575	3	5	2	5	12	0	1	1
SY576	1	5	4	5	14	0	0	0
SY577	4.3	5	5	5	15	0	4	4
SY578	5	2	3	0	5	0	4	4
SY579	5	0	5	3.5	8.5	0	0	0
SY580	2	0	0	0	0	5	0	5
SY583	5	5	5	5	15	5	0	5
T500	2	0	1	1	2	0	0	0
T505	3	0	0	2	2	0	2	2
T506	2.5	0	0	0	0	0	0	0
T508	2	0	2	2	4	0	0	0
T510	1	2	2	0	4	2	0	2
T511	1	0	1	2	3	0	0	0
T514	3.5	0	1.5	1	2.5	0	0	0
T515	2.5	3	0	3	6	0	0	0
T525	3.5	0	0	0	0	0	0	0
T535	5	4.2	5	5	14.2	3.0	2.8	5.8

ML, Mexican lime (Citrus aurantifolia Christm. Swing.); SO, sour orange (C. aurantium L.), Le, Eureka or Lisbon lemon (C. limon L. Burm.f.); GF, Duncan grapefruit (C. paradisi Macf.), and SW, Madam Vinous or Pineapple sweet orange (C. sinensis)0.0–5: Symptoms evaluation scale. 0 = negative; 1 = very mild; 2 = mild; 3 = moderate; 4 = severe; and 5 = very severe.

The biological characterization data were analyzed in relation to the location (SC, CC, and XC), period (1 and 2), type of original source (CES/CCPP introductions, urban/commercial groves, and CPDP interceptions), and genotype (T30, T36, and VT) of the CCPP CTV isolates (Tables 1, 4). Geographical location, time period, source type, genotype and CP gene phylogeny of the CTV isolates had no significant effect on the Mexican lime reactions (*p* > 0.05, ANOVA). In contrast, XC isolates had significantly higher biological activity scores than CC isolates (*F*_30_ = 3.656, *p* = 0.039). Similarly, CTV isolates from period 1 induced more severe biological reactions than those from period 2 (*F*_30_ = 5.802, *p* = 0.023). When geographical location and time period were co-analyzed, CTV isolates SC-1 and XC-2 induced significantly more severe biological reactions than SC-2 and CC-2 (*F*_30_ = 6.324, *p* = 0.002). CTV isolates from CES/CCPP (type A) were significantly more virulent than the isolates naturally spreading in urban and commercial groves (type B) (*F*_30_ = 5.008, *p* = 0.014). CTV isolates with T30 genotype induced significantly milder biological activity scores in comparison to VT and T30+VT+T36 (*F*_30_ = 3.123, *p* = 0.025). The milder biological activity of T30 genotypes compared to VT genotypes was also supported by the significantly different biological score of CTV isolates that clustered in clade 5 (VT representatives) versus clade 1 (T30 representatives) (*F*_30_ = 10.014, *p* < 0.001).

SY and SP severity scores were significantly correlated with time periods and geographical location of the CTV isolates, respectively. Period 1 isolates induced significantly more severe SY (*H*_1_ = 6.165, *p* = 0.013) and XC isolates induced significantly more severe SP (*H*_2_ = 7.636, *p* = 0.022). Spatiotemporal analysis of the SY and SP reactions indicated significantly higher SY reactions for SC-1 isolates in comparison to SC-2 and CC-2 (*F*_30_ = 5.033, *p* = 0.007) and significantly higher SP reaction for XC-2 in comparison to SC-1 and -2 and CC-2 (*H*_3_ = 8.035, *p* = 0.045). CTV isolates clustered in clade 5 (VT representative) had significantly more severe SY reactions in comparison to isolates from clade 1 (T30 representative) (*F*_27_ = 8.297, *p* < 0.001). CTV source types and genotypes had no significant effects in SY and SP reactions (*p* > 0.05).

## Discussion

The one-of-a-kind century-old *in planta* CCPP CTV collection has proven valuable for CTV research. From the early days of CTV discovery, detection, and biological characterization to today's molecular era, the collection has provided important information for the virus in California (Gumpf et al., 1987; Garnsey et al., 1991; Marco and Gumpf, 1991; Roistacher, 1991; Nikolaeva et al., 1998; Vidalakis et al., 2004; Wang et al., 2012). We understand that the data developed in this report is based on a relatively small sample size to reach any definitive or general conclusions for the genotype or evolutionary relationships of CTV isolates in California. However, each isolate tested was often a lone selection among many isolates detected as a representative of location, time, host combination, symptomology, etc. Furthermore, the statistical analysis served to normalize data from unequal or non-uniform CTV samples in the different categories tested.

The frequencies of CTV genotypes and biological characterization of the isolates from the past 100 years, different location, and source types in California were consistent with that expected due to selected citrus introductions [e.g., selection against stem pitting (SP) isolates], eradication and suppression efforts (e.g., reduced genotype diversity in period 2 and CPDA interceptions), and use of virus-tested stock (e.g., reduced genotype diversity in CC-2) (Roistacher et al., 1981; Yokomi and Deborde, 2005; Barnier et al., 2010). If citrus germplasm were introduced freely in California (i.e., without CTV testing and elimination) several CTV genotypes would be expected to be present in the state. Moreover, CTV association with aphid vectors over time would have created more genotype combinations in California. This study revealed only three of the five known CTV genotypes. Furthermore, 90% of the isolates had T30 genotypes in single and double infections with VT genotypes. Even though factors such as transmission efficiency of different genotypes or other molecular evolutionary events (mutations, recombination, etc.) could have affected our results, the data agrees with and provides support to previous reports identifying T30 and VT as the most common CTV genotypes in California (Yokomi and Deborde, 2005; Yokomi et al., 2011a).

Many CCPP CTV isolates were mixtures of multiple genotypes thus; it is difficult to ascertain which genotypes present directly induced the observed SP and SY symptoms. Interestingly, less than half of the CCPP CTV isolates induced SP with low symptom severity. The opposite was observed for SY. Almost all CTV isolates induced SY reactions with the majority being severe. SP is expressed in the field on various citrus scion species but not SY. SY is a greenhouse indicator reaction that is not expressed in field trees. This supports the hypothesis that early citrus researchers, nurserymen and growers selected citrus planting stock for California from non-SP symptomatic vigorous trees. In contrast, the SY phenotype would have been undetectable in the field and it would have required biological indexing for identification. Thus, selection of citrus stock from the 1800's to the 1950's would have no way to be tested for CTV SY and, as a result, SY isolates were likely unwittingly allowed to pass into California.

The diverse phylogenetic relationships and increased virulence of XC and SC CTV isolates should be expected as they represent the earliest arrival of CTV populations before any controls were imposed by certification and eradication programs. This was evident from the limited phylogenetic relationships and genotype diversity of the CC CTV isolates compared to SC isolates due to the benefits of the fore-mentioned control measures implemented in California (Roistacher, 1995; Gottwald et al., 2002; Barnier et al., 2010).

The CCPP CTV collection also provided a combination of biological and molecular isolates to evaluate performance of CTV detection/characterization tools. Mexican lime is well known and widely used CTV indicator host. The data presented here provided experimental evidence and statistical support that Mexican lime can be considered as the standard method for broad-spectrum CTV detection since it reacted with all CTV isolates tested regardless of genotype, origin, etc. In addition, the MMM protocol as described by Hilf et al. (2005) and Roy et al. (2010) readily identified older as well as recent isolates with single CTV genotypes T30 and VT. In contrast, identification of the T3 genotype and various other genotype mixtures were problematic. It is likely that recombination events, especially in mixed CTV infections, contributed to the observed irregular MMM results (Vives et al., 1999; Hilf et al., 2005; Vives et al., 2005; Moreno et al., 2008; Roy and Brlansky, 2010).

Our study also highlighted the need to use complimentary analysis by different methodologies of CTV characterization. For example, MMM analysis did not identify B165/T68 genotypes and the identification of T3 genotype was inconclusive for the CCPP CTV isolates. However, use of CP gene sequencing and phylogeny suggested relationships of CCPP CTV isolates with T3 and B165/T68 genotypes. In addition, the MMM analysis identified T30 genotypes in CTV isolates that induced severe SY reaction in plant indicators. While T30 genotypes are not known to produce SY, it is possible, that other genotypes that were not identified by the utilized MMM protocols were present and responsible for the observed reactions. Specific genome regions, such as p23, of the CCPP CTV T30 isolates that induced SY reactions should also be further investigated (Albiach-Marti et al., 2010). Regardless of the mechanism behind the association of T30 genotypes with SY reactions, the present study indicated that in the absence of any biological information, isolates such as SY563 could have been considered benign based on genotype information alone. In our case, the combination of different methodologies provided an opportunity for careful interpretation of the molecular data as well as testable hypotheses for further experimentation with specific CTV isolates.

California has been fortunate so far in avoiding introduction of exotic CTV isolates such as A18, Taiwan-Pum/SP/T1 (JX266712), and NUagA and eradicating virulent CTV-SP isolates, such as SY568, SY553 (Meyer lemon) and T535 (Dekopon), before they could spread to commercial citrus (Calavan et al., 1980; Roistacher and Dodds, 1993; Vives et al., 1999; Gottwald et al., 2002; Moreno et al., 2008; Herrera-Isidrón et al., 2009; Ruiz-Ruiz et al., 2009; Roy et al., 2010; Saponari and Yokomi, 2010). Our genotype, phylogenetic, and biological analysis provided useful information for monitoring of CA-exotic CTV isolates, development of diagnostics and management strategies such as CTV-SP cross-protection (Roistacher et al., 1988; Roistacher and Dodds, 1993; Karasev, 2000; Folimonova et al., 2010; Folimonova, 2012; Matos et al., 2013).

### Conflict of interest statement

The authors declare that the research was conducted in the absence of any commercial or financial relationships that could be construed as a potential conflict of interest.
